# Upper Gastrointestinal Bleeding from Gastric Amyloidosis in a Patient with Smoldering Multiple Myeloma

**DOI:** 10.1155/2015/320120

**Published:** 2015-08-23

**Authors:** Mihajlo Gjeorgjievski, Treta Purohit, Mitual B. Amin, Paul J. Kurtin, Mitchell S. Cappell

**Affiliations:** ^1^Department of Internal Medicine, William Beaumont Hospital, Royal Oak, MI 48073, USA; ^2^Division of Gastroenterology & Hepatology, William Beaumont Hospital, Royal Oak, MI 48073, USA; ^3^Anatomic Pathology, William Beaumont Hospital, Royal Oak, MI 48073, USA; ^4^Oakland University William Beaumont School of Medicine, Royal Oak, MI 48073, USA; ^5^Laboratory Medicine and Pathology, Mayo Clinic, Rochester, MN 55905, USA

## Abstract

Amyloidosis is a common complication of patients with monoclonal gammopathy of undetermined significance (MGUS), smoldering multiple myeloma (SMM), and multiple myeloma (MM). This proteinaceous material can be deposited intercellularly in any organ system, including the gastrointestinal (GI) tract. In the GI tract, amyloidosis affects the duodenum most commonly, followed by the stomach and colorectum. Gastric amyloidosis causes symptoms of nausea, vomiting, early satiety, abdominal pain, and GI bleeding. A case of upper GI bleeding from gastric amyloidosis is presented in a patient with SMM. Esophagogastroduodenoscopy (EGD) revealed a gastric mass. Endoscopic biopsies revealed amyloid deposition in the lamina propria, consistent with gastric amyloidosis. Liquid chromatography tandem mass spectrometry performed on peptides extracted from Congo red-positive microdissected areas of paraffin-embedded stomach specimens revealed a peptide profile consistent with AL- (lambda-) type amyloidosis. Based on this and multiple other case reports, we recommend that patients with GI bleeding and MGUS, SMM, or MM undergo EGD and pathologic examination of endoscopic biopsies of identified lesions using Congo red stains for amyloidosis for early diagnosis and treatment.

## 1. Introduction

Patients with monoclonal gammopathy of undetermined significance (MGUS) and smoldering multiple myeloma (SMM) are at high risk to develop multiple myeloma (MM) and amyloidosis. The amyloidosis can infiltrate various organs, including any part of the gastrointestinal (GI) tract. Common symptoms of GI amyloidosis include GI bleeding, nausea, vomiting, and chronic abdominal pain [1–3]. Such GI symptoms in patients with known MGUS, SMM, or MM should prompt consideration of amyloidosis in the differential diagnosis, performance of GI endoscopy with biopsy, and special stains for amyloidosis on the endoscopic biopsies because the diagnosis of amyloidosis can alter the treatment and prognosis. A case is reported of GI bleeding associated with SMM, with GI amyloidosis diagnosed by esophagogastroduodenoscopy (EGD), Congo red stains performed on endoscopic biopsies, and liquid chromatography tandem mass spectrometry performed on peptides extracted after microdissection of Congo red-positive areas.

## 2. Case Report

A 92-year-old woman with a history of SMM presented with progressive weakness, lethargy, orthostatic dizziness, and melena during the prior few days. Bone marrow biopsy performed three months earlier had revealed a variably cellular marrow with trilineage hematopoiesis. The bone marrow core biopsy and clot section contained numerous plasma cells, frequently in small clusters and in a few large aggregates, accounting for nearly 50% of the total cellularity. The plasma cells were small in size, were predominantly mature in morphology, and occasionally included atypical forms with slightly irregular nuclear contours and increased nuclear-to-cytoplasmic ratio. Immunohistochemical stains performed on the core biopsy revealed lambda light-chain restriction of CD138+ plasma cells and rare kappa+ plasma cells. A Congo red stain was not performed as part of the bone marrow biopsy. Serum immunoglobulin monoclonal protein (IgG lambda M-protein) was 2.2 mg/dL. A 24-hour urine collection revealed 0.01 g/24 hours of albumin, with 0.07 g/24 hours of monoclonal free Lambda light chain (Bence-Jones proteins) as demonstrated by immunofixation. There was no evident end-organ disease, including absence of hypercalcemia, anemia, or renal failure. A complete bone survey revealed no lytic lesions.

**Figure 1:**
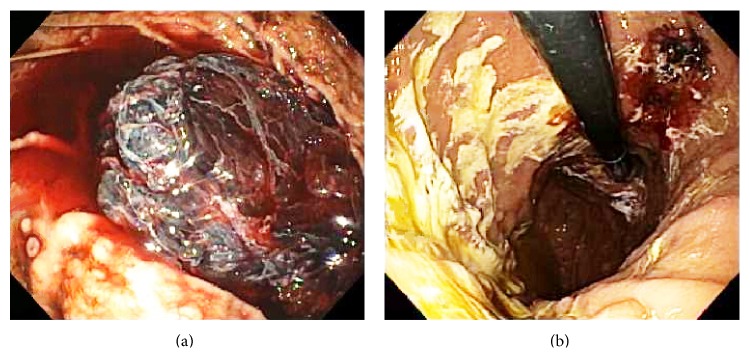
Initial EGD performed for an acute episode of melena in a 92-year-old woman with smoldering multiple myeloma (SMM) revealed a well-organized, hemispherical, 11 × 6 cm tightly adherent clot attached to the midgastric body that that could not be detached despite vigorous endoscopic irrigation and aspiration (a). About eight, 3–5 mm wide sessile polyps (nodules) were present in the gastric fundus and body, devoid of stigmata of recent hemorrhage, two of which were present at the 8 o'clock position. Repeat EGD performed one day later revealed that the clot had mostly dissolved exposing a 2.5 × 2 cm ulcerated mass, mostly covered by a clot in the midgastric body along the lesser curvature (b).

**Figure 2:**
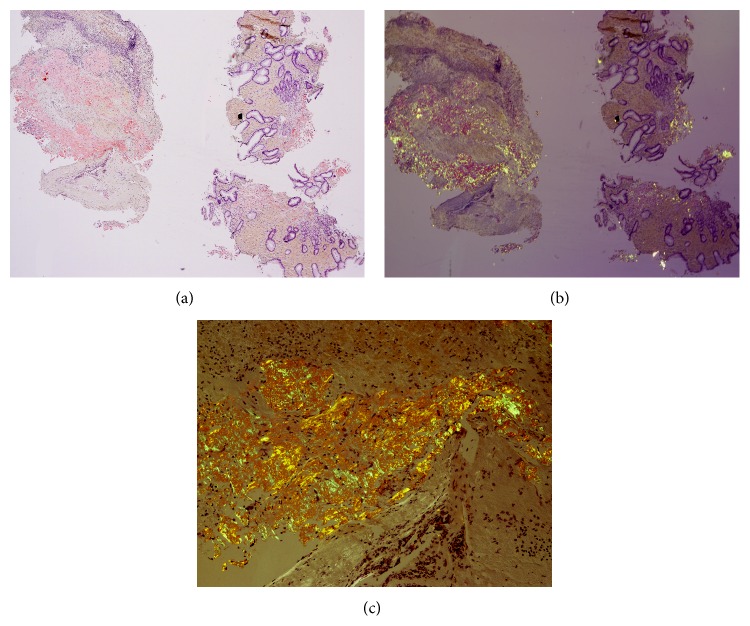
Low-power photomicrograph of endoscopic biopsies of the gastric mass using a Congo red stain revealed rose-pink staining of the amyloid deposits (a). Low-power (b) and high-power (c) photomicrographs of Congo red stain from the same sections using polarized light revealed the classic apple-green birefringence. Note that the regions exhibiting apple-green birefringence in (b) and (c) correspond to the same regions revealing rose-pink staining in (a). Note the presence of typical gastric glands on the right sides of (a) and (b).

**Figure 3:**
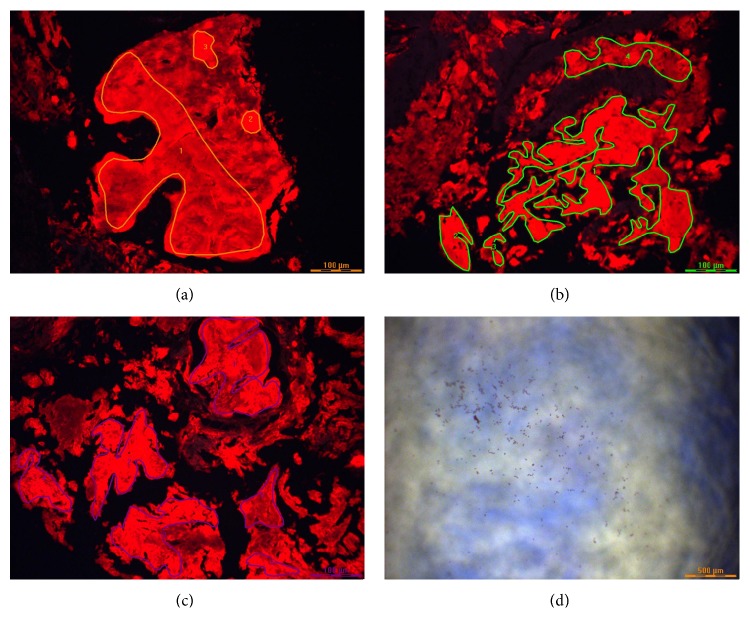
Congo red-positive sections viewed under ultraviolet light in the microdissection microscope. The amyloid fluoresces red. The drawn lines (yellow in (a), green in (b), and blue in (c)) delineate areas circumscribing the amyloid deposits that were cut out of the tissue sections for processing for mass spectrometry. The three dissections in (a)–(c) correspond to the three patient sample columns in the Scaffold illustration in Table 1. Each sample was processed in triplicate. (d) shows the amyloid fragments that have been cut out of the tissue and are now in the reaction cup for further processing (i.e., reduction of disulfide bonds, trypsin digestion, etc.) before mass spectrometry.

Physical examination on admission revealed normal vital signs, a soft and nontender abdomen, and melena on rectal examination. The hematocrit was 32.5% (normal in females: 35.4%–44.2%). EGD revealed a large, hemispherical, 11 × 6 cm adherent blood clot attached to the gastric body that could not be detached despite vigorous endoscopic irrigation and aspiration (Figure 1(a)). About eight small, sessile, polyps were present in the gastric fundus and body without stigmata of recent hemorrhage (SRH). No lesions were present in the esophagus or duodenum. The patient was administered intravenous pantoprazole at 8 mg/hr after an 80 mg loading dose and transfused 2 units of packed erythrocytes. Repeat EGD performed one day later revealed that the clot had mostly dissolved exposing a not actively bleeding, 2.5 × 2 cm ulcerated mass, mostly covered by clot in the midgastric body along the lesser curvature (Figure 1(b)). Histologic staining with Congo red stain revealed positively staining deposits in the lamina propria (Figure 2(a)) that exhibited apple-green birefringence under polarized light (Figures 2(b) and 2(c)), consistent with gastric amyloidosis. Peptides were extracted from Congo red-positive areas after microdissection of paraffin-embedded stomach biopsy specimens (Figure 3); and then liquid chromatography tandem mass spectrometry (LC MS/MS) was performed on the extracted peptides which revealed a peptide profile consistent with AL- (lambda-) type amyloidosis (Table 1).

Table 1 includes many of the proteins identified in the specimen as displayed by the Scaffold software. “Starred” entries are amyloid-associated proteins placed at the top of the list for ease of identification. Numbers marked by asterisks correspond to numbers in the mass spectrometry (MS) spectra for each identified protein. The asterisk over the spectral count represents the probability that the corresponding protein has been correctly identified. Spectral counts ≥ 5 have *p* > 95%. Spectral counts < 5 are not interpreted because *p* < 95%. Columns titled* Patient Samples 1*,* 2*, or* 3* correspond to results from the 3 microdissections performed in Figure 3 (i.e., all cases analyzed by LC-MS/MS in triplicate). The samples contain abundant apolipoprotein A-IV, apolipoprotein E, and serum amyloid P-component peptides that are all codeposited in all types of amyloid. Additionally, all three replicate analyses contained abundant lambda light chain, which is the specific amyloid protein in this patient (i.e., the specimen contains amyloidosis, AL-lambda light chain-type).

Other immunoglobulin proteins (gamma, alpha, and kappa) represent proteins in the background interstitial fluid/serum. This finding is demonstrated by these proteins having much lower spectral counts than the lambda protein and by the presence in the sample of considerable albumin, a marker for “serum contamination.” All the other listed proteins are typical tissue constituents, except for trypsin which was added to the sample to digest the proteins prior to LC-MS/MS analysis. The authors thank Jason D. Theis, B.S., in the Department of Laboratory Medicine and Pathology, at the Mayo Clinic, Rochester, Minnesota, for technical help in identifying the proteins in the specimen.

The patient had no further episodes of GI bleeding and was discharged home after 17 days in the hospital while receiving omeprazole 40 mg orally once daily. No bleeding has been noted during one-month follow-up.

## 3. Discussion

SMM is an asymptomatic proliferative disorder of plasma cells that can progress to MM. It is differentiated from MM by absence of end-organ damage. SMM is far more likely to progress to MM or amyloidosis than MGUS (78% versus 21% risk of progression) [4]. Amyloidosis is a condition of extracellular fibrillar protein deposition, that is, commonly associated with tissue injury and dysfunction [5]. Primary amyloidosis usually represents amyloidosis associated with immunocyte dyscrasia from monoclonal proliferation of plasma cells that synthesize an immunoglobulin that is prone to form amyloid.

GI amyloidosis can occur as an isolated entity or as part of multisystem involvement. Different types of amyloid proteins can deposit in various parts of the GI tract or liver, manifesting as abdominal pain, GI dysmotility, diarrhea, GI bleeding, or hepatic injury [1]. Suspected GI amyloidosis, irrespective of underlying etiology, requires biopsies of affected tissue [6]. GI involvement is uncommon in patients with amyloidosis, with a reported risk of only about 3% [6]. Among patients with GI involvement, 79% have underlying systemic amyloidosis, and 21% have only GI amyloidosis without evident plasma cell dyscrasia or extraintestinal involvement. Most systemic cases have underlying immunoglobulin light-chain disease (83%) [6]. GI bleeding was the second most frequent symptom of amyloidosis (36%), after weight loss (45%), and closely followed by heartburn (33%) [6]. Congo red stains are recommended for patients with unexplained weight loss, GI bleeding, abdominal pain, or early satiety associated with a monoclonal gammopathy [6]. In a case series of 37 cases of GI amyloidosis, the relative frequency of amyloid deposition was 100% in the duodenum, 95% in the stomach, and 91% in the colorectum [7].

In patients with primary systemic amyloidosis, Menke et al. [8] reported an 8% prevalence of GI involvement, with only 1% having symptomatic gastric involvement. Common presenting symptoms with gastric amyloidosis included hematemesis, prolonged nausea and vomiting, gastroparesis, or gastric outlet obstruction. Diagnosis of GI amyloidosis without previously diagnosed inflammatory or plasma cell disorders is exceptionally rare [9]. In one case report, it has been proposed that amyloidosis should always be considered in the differential diagnosis of hematemesis and gastric tumors [10]. In another case report, a strong suspicion of amyloidosis was recommended in patients with multiple myeloma and obscure GI bleeding [2].

The endoscopic appearance of gastric amyloidosis can closely resemble that of gastric malignancy [10]. Amyloidosis can appear as submucosal tumors, polyps, antral narrowing, thickened irregular gastric folds, or loss of rugal folds. Other appearances include gastric ulcers, hematomas, arteriovenous malformations, granular-appearing mucosa, plaque-like lesions, or ulcerative gastritis, often associated with GI bleeding [3].

The pathophysiology of GI bleeding from amyloidosis involves local ischemia, infarction, and mucosal injury that cause erosions, hematomas, or ulcerations. When GI bleeding is the presenting symptom, endoscopy commonly reveals a submucosal hematoma [7, 11]. GI bleeding caused by gastric amyloidosis may be obscure or overt. Hematoma rupture in patients with gastric amyloidosis can sometimes cause life-threatening, GI bleeding. Currently, no treatment guidelines exist for endoscopic therapy for bleeding from gastric amyloidosis, and the decision on which endoscopic therapy is used is based on the type of lesion present and the endoscopist's preference. Endoscopic hemostasis is often ineffective and surgical intervention may be required [12]. In the currently reported patient endoscopic therapy was not performed because there was no active source of bleeding during repeat EGD. The patient was placed on intravenous proton pump inhibitor initially which was later switched to omeprazole 40 mg twice a day. There was no recurrent bleeding during one month of follow-up.

## Figures and Tables

**Table 1:** 

#	Starred	Bio View: identified proteins (649)	Accession number	Molecular weight	Patient sample 1	Patient sample 2	Patient sample 3
1	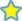	Ig lambda-2 chain C regions	LAC2_HUMAN	11 kDa	31^*∗*^	21^*∗*^	34^*∗*^
2	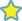	Apolipoprotein A-IV	APOA4_HUMAN	45 kDa	28^*∗*^	17^*∗*^	15^*∗*^
3	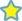	Apolipoprotein E	APOE_HUMAN	36 kDa	21^*∗*^	11^*∗*^	15^*∗*^
4	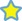	Serum amyloid P-component	SAMP_HUMAN	25 kDa	22^*∗*^	13^*∗*^	13^*∗*^
5	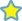	Ig gamma-1 chain C region	IGHG1_HUMAN	36 kDa	6^*∗*^	10^*∗*^	7^*∗*^
6	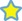	Ig alpha-1 chain C region	IGHA1_ HUMAN	38 kDa	6^*∗*^	5^*∗*^	
7	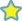	Ig gamma-3 chain C region	IGHG3_HUMAN	41 kDa	4^*∗*^	7^*∗*^	
8	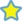	Ig kappa chain C region	IGKC_HUMAN	12 kDa	4^*∗*^	3^*∗*^	
9	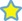	Ig kappa chain V-III region…	KV302_HUMAN…	12 kDa	4^*∗*^		
10	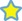	Ig lambda chain V-II region…	LV205_HUMAN	12 kDa		3^*∗*^	
11		(ENZYME) trypsin precursor	ENZYME_TRYP_…	24 kDa	230^*∗*^	223^*∗*^	220^*∗*^
12		Vitronectin	VTNC_HUMAN	54 kDa	38^*∗*^	31^*∗*^	40^*∗*^
13		Keratin, type II cytoskeletal…	K2C1_HUMAN	66 kDa	6^*∗*^	49^*∗*^	20^*∗*^
14		Keratin, type I cytoskeletal…	K1C10_HUMAN	59 kDa	7^*∗*^	35^*∗*^	12^*∗*^
15		Apolipoprotein A-I	APOA1_HUMAN	31 kDa	20^*∗*^	20^*∗*^	5^*∗*^
16		Serum albumin	ALBU_HUMAN	69 kDa	8^*∗*^	15^*∗*^	15^*∗*^
17		Keratin, type II cytoskeletal…	K22E_HUMAN	65 kDa		33^*∗*^	1^*∗∗*^
18		Hemoglobin subunit alpha	HBA_HUMAN	15 kDa	6^*∗*^	3^*∗∗*^	24^*∗*^
19		Complement component C9	CO9_HUMAN	63 kDa	16^*∗*^	16^*∗*^	
20		Keratin, type I cytoskeletal 9	K1C9_HUMAN	62 kDa		21^*∗*^	10^*∗*^
21		Hemoglobin subunit beta	HBB_HUMAN	16 kDa	6^*∗*^	2^*∗*^	20^*∗*^
22		Plasminogen	PLMN_HUMAN	91 kDa	15^*∗*^	13^*∗*^	1^*∗∗*^
23		Fibrinogen alpha chain	FIBA_HUMAN	95 kDa			31^*∗*^
24		Fibrinogen beta chain	FIBB_HUMAN	56 kDa			27^*∗*^
25		Fibrinogen gamma chain	FIBG_HUMAN	52 kDa			25^*∗*^
26		Collagen alpha-1(I) chain	CO1A1_HUMAN	139 kDa	6^*∗*^	1^*∗∗*^	12^*∗*^
27		Collagen alpha-2(I) chain	CO1A2_HUMAN	129 kDa	11^*∗*^		8^*∗*^
28		Trypsin-3	TRY3_HUMAN	33 kDa	4^*∗*^	4^*∗*^	4^*∗*^
29		Collagen alpha-3(VI) chain	CO6A3_HUMAN	344 kDa	2^*∗*^	6^*∗*^	8^*∗*^
30		395 ribosomal protein L40,…	RM40_HUMAN	24 kDa	3^*∗∗*^	2^*∗∗*^	4^*∗∗*^

Probability legend: ^*∗*^over 95%; ^*∗∗*^80% to 94%.
